# Anticoagulant treatment satisfaction with warfarin and direct oral anticoagulants for venous thromboembolism

**DOI:** 10.1007/s11239-021-02437-z

**Published:** 2021-04-08

**Authors:** Margaret C. Fang, Alan S. Go, Priya A. Prasad, Jin-Wen Hsu, Dongjie Fan, Cecilia Portugal, Sue Hee Sung, Kristi Reynolds

**Affiliations:** 1 Division of Hospital Medicine, University of California, San Francisco, 521 Parnassus Ave., Box 0131, San Francisco, CA 94143 USA; 2 Division of Research, Kaiser Permanente Northern California, Oakland, CA USA; 3 Department of Health Systems Science, Kaiser Permanente Bernard J. Tyson School of Medicine, Pasadena, CA USA; 4 Department of Medicine and Department of Epidemiology and Biostatistics, University of California, San Francisco, San Francisco, CA USA; 5 Departments of Medicine, Health Research and Policy, Stanford University, Palo Alto, CA USA; 6 Department of Research and Evaluation, Kaiser Permanente Southern California, Pasadena, CA USA

**Keywords:** Anticoagulants, Venous thromboembolism, Treatment satisfaction, Direct oral anticoagulants, Warfarin

## Abstract

Treatment options for patients with venous thromboembolism (VTE) include warfarin and direct oral anticoagulants (DOACs). Although DOACs are easier to administer than warfarin and do not require routine laboratory monitoring, few studies have directly assessed whether patients are more satisfied with DOACs. We surveyed adults from two large integrated health systems taking DOACs or warfarin for incident VTE occurring between January 1, 2015 and June 30, 2018. Treatment satisfaction was assessed using the validated Anti-Clot Treatment Scale (ACTS), divided into the ACTS Burdens and ACTS Benefits scores; higher scores indicate greater satisfaction. Mean treatment satisfaction was compared using multivariable linear regression, adjusting for patient demographic and clinical characteristics. The effect size of the difference in means was calculated using a Cohen’s *d* (0.20 is considered a small effect and ≥ 0.80 is considered large). We surveyed 2217 patients, 969 taking DOACs and 1248 taking warfarin at the time of survey. Thirty-one point five percent of the cohort was aged ≥ 75 years and 43.1% were women. DOAC users were on average more satisfied with anticoagulant treatment, with higher adjusted mean ACTS Burdens (50.18 v. 48.01, p < 0.0001) and ACTS Benefits scores (10.21 v. 9.84, p = 0.046) for DOACs vs. warfarin, respectively. The magnitude of the difference was small (Cohen’s *d* of 0.29 for ACTS Burdens and 0.12 for ACTS Benefits). Patients taking DOACs for venous thromboembolism were on average more satisfied with anticoagulant treatment than were warfarin users, although the magnitude of the difference was small.

**Supplementary Information:**

The online version contains supplementary material available at 10.1007/s11239-021-02437-z.

## Highlights

- Few studies have compared whether patients with venous thromboembolism are more satisfied on direct oral anticoagulants (DOACs) or warfarin
- We surveyed 2217 adults taking oral anticoagulants for venous thromboembolism and assessed anticoagulant treatment satisfaction using a validated scale (the Anti-Clot Treatment Scale)
- Average treatment satisfaction on DOACs was higher than on warfarin, although the magnitude of the difference was small
- Whether treatment satisfaction leads to differential medication adherence or patient behavior is not known.

## Background

Venous thromboembolism (VTE) affects an estimated 500,000 individuals each year in the United States [1]. The mainstay of primary treatment for VTE is therapeutically-dosed anticoagulation, generally for a period of 3–6 months, followed by consideration of further anticoagulation depending on the clinical context [2, 3]. However, anticoagulants can lead to both life-threatening and non-life threatening bleeding, as well as cause patients to alter their lifestyle and behaviors; these could negatively impact quality of life. Oral anticoagulant options for VTE include vitamin K antagonists, most commonly warfarin sodium, and more recently, several direct oral anticoagulants (DOACs) including dabigatran, rivaroxaban, apixaban, and edoxaban. DOACs have advantages over warfarin in terms of ease of use: they have fixed dosing, do not require frequent blood tests for monitoring, and are associated with fewer drug-drug and dietary interactions [4]. However, DOACS are more costly than warfarin, and like all anticoagulants, can increase the risk of bleeding. When selecting amongst various anticoagulant options, clinicians must weigh efficacy and safety, whether a patient can adhere to a treatment plan, and cost. In addition to clinical outcomes, patients may also prioritize convenience and ease of use.

Relatively few studies that have compared treatment satisfaction between DOACs and warfarin users in patients with VTE, particularly in non-clinical trial settings [5, 6]. This study’s goal was to measure anticoagulant treatment satisfaction in a large, community-based cohort of patients anticoagulated for VTE, comparing patients taking warfarin with patients taking DOACs.

## Methods

### Participants and setting

The source population for the study was adults (aged ≥ 18 years) enrolled in Kaiser Permanente Northern California (KPNC) or Kaiser Permanente Southern California (KPSC), two large, integrated healthcare delivery systems providing comprehensive care for approximately 9 million individuals across California. The source population is representative of the racially and ethnically diverse population of California [7, 8].

We identified all adults who completed an initial treatment course (e.g., 3 months) of oral anticoagulants after an incident diagnosis of acute VTE that occurred during the time period between January 1, 2010 and December 31, 2018. Incident diagnosis of VTE was defined as a clinical encounter (inpatient, emergency department, or outpatient) associated with either a primary or secondary discharge diagnosis of VTE according to the International Classification of Diseases Ninth Revision (ICD-9) or Tenth Revision (ICD-10), and with no prior VTE diagnosis or oral anticoagulant prescription in the past 4 years. The cohort was further restricted to individuals with complete information on age and gender, and with 12 months of continuous enrollment and pharmacy benefits prior to the VTE index date. VTE type was categorized as pulmonary embolism (PE) with or without other thrombosis, lower extremity deep venous thrombosis (DVT), upper extremity DVT, and other VTE (such as mesenteric thrombosis). Anticoagulant prescriptions were identified from health plan pharmacy dispensing databases. Data on subject demographics (age, self-reported gender, race, and Hispanic ethnicity) were obtained from health plan administrative databases.

We identified and invited a set of eligible patients to participate in a survey on anticoagulant treatment satisfaction. Eligibility criteria were non-deceased patients with a VTE diagnosis date between January 1, 2015 and June 30, 2018 (to capture the experience of patients once there was more widespread use of DOACs for VTE), actively enrolled in the health plan, who had a valid mailing address and/or telephone number at the time of the survey, and whose primary language was English, Spanish, or Chinese. Patients were categorized as either taking warfarin or a DOAC (dabigatran, rivaroxaban, or apixaban), using the anticoagulant prescription closest in time to the survey completion date.

Surveys were delivered by ANA Research, Inc., a market research company, and occurred in two waves, the first during Summer, 2018 and the second in Spring, 2019. Eligible subjects were mailed a survey invitation and, when possible, sent an email invitation as well. Follow-up phone calls were issued to non-respondents. Subjects were given the opportunity to complete the survey in English, Spanish, or Chinese. Surveys could be completed on a paper form, by telephone, or online via secure web link. An honorarium of US $25 was offered upon survey completion. In the first survey wave, we contacted all DOAC and warfarin users with an index VTE between January, 2015 and April, 2017 to participate in web, telephone, or paper versions of the survey. In the second survey wave, patients with VTE diagnosis between May, 2017 and June, 2018 were invited to complete the web survey. To ensure adequate representation of DOAC users, we then mailed survey invitations to DOAC users who did not have a valid e-mail address or if they did not respond to the e-mail survey link.

The survey asked patients whether they took anticoagulants within the past 4 weeks and if yes, to complete the Anti-Clot Treatment Scale© (ACTS) [9], a validated 17 item patient-reported scale that assesses treatment satisfaction with anticoagulants. Additionally, questions included whether they had ever switched from one anticoagulant to another, and had they ever experienced bleeding complications from anticoagulants. Finally, the survey asked about subjects’ marital status, household income, and highest educational attainment.

### Statistical analysis

The primary outcome of this analysis was anticoagulant treatment satisfaction measured by the ACTS. The ACTS is divided into two sub-scales, ACTS Burdens (assessing challenges with anticoagulation) and ACTS Benefits (assessing confidence and reassurance in anticoagulation). In addition, there are two global questions asking about overall benefit and overall burden which are not included in the scores. ACTS Burdens is reverse coded on a 5-point Likert scale and is the sum of 12 items (range 12–60). ACTS Benefits is coded from 1 to 5 and is the sum of 3 items (range 3–15). Higher scores denote greater satisfaction with treatment. We calculated the ACTS scores according to the developers’ guidelines [9]. If > 50% of items were not answered by a subject, the participant’s response was considered missing; otherwise individual subscale imputation to the mean was used for missing items.

We first compared the unadjusted mean treatment satisfaction (ACTS Burdens and ACTS Benefits) between patients on warfarin and DOACs. In addition to statistically significant difference, we also calculated the effect size, a measure of the size of the difference in outcomes that can inform the clinical relevance of the treatment difference [10]. Effect size was calculated as the Cohen’s *d*, the difference in means divided by the pooled standard deviation. In general, an effect size of 0.20 is considered small, an effect size of 0.50 is moderate, and effect sizes of ≥ 0.80 are large.

Next, we developed separate general multivariable linear regression models for ACTS Burdens and ACTS Benefits scores, with the primary independent variable being anticoagulant treatment category (warfarin versus DOAC), and adjusted for clinical and demographic characteristics that may be plausible confounders of the relationship between treatment type and satisfaction. The difference in mean satisfaction scores were computed as the Least Squares mean, adjusted for the means of the other covariates. For race and ethnicity, a separate category of “missing/unknown” was included. Most other variables had minimal to no missing data, so multivariable models were developed from those subjects with complete data. There was no missing data for the primary independent variable, anticoagulant treatment category. All models also included a high-dimensional propensity score (hdPS) that represented the likelihood a patient was prescribed a DOAC. In contrast to standard propensity score development, which is typically based on a set of pre-selected variables, a hdPS is developed through automated or data-driven algorithms that take advantage of the availability of large administrative data sources and may approximate point estimates of risk more accurately than standard propensity scores [11]. The hdPS for this study was developed using 5 dimensions (principal inpatient/emergency department [ED] diagnoses only, secondary inpatient/ED diagnoses, outpatient/ED diagnoses, procedures from any setting, and outpatient drug claims) and used a look-back period of 4 years. Within each dimension, the 300 most common codes were ranked by frequency, and then the final hdPS was developed by regressing the exposure variable (anticoagulation treatment type) on the covariates. The hdPS was then included as an adjustment variable in the multivariable models.

Finally, because patients who change anticoagulant treatment types may have done so due to dissatisfaction with a given treatment, we repeated the analyses restricting to patients who did not report a history of switching anticoagulants.

This study was approved by the Kaiser Permanente Northern California Institutional Review Board. The authors had full access to all the data in the study and take responsibility for its integrity and the data analysis.

## Results

Out of 12,737 eligible patients approached to participate in the original survey, 4771 completed at least 80% of the items and 2244 people answered at least 1 item on the ACTS instrument (not all subjects who answered the survey reported actively taking anticoagulants and so were not eligible for the treatment satisfaction portion of the survey). Twenty-seven people answered < 50% of the ACTS items and were excluded from the analysis, resulting in a final analytic population of 2217 patients, of whom 969 were taking a DOAC and 1248 were taking warfarin. Similar proportions of warfarin and DOAC users completed the ACTS instrument, with 98.2% of warfarin users answering ≥ 90% of the ACTS items and 98.8% of DOAC users (p = 0.26).

Compared with warfarin users, patients taking DOACs were on average younger and with higher educational attainment and household income (Table 1). Patients taking DOACs were more likely to have been diagnosed with their index VTE in recent years, reflecting increased uptake of DOACs over time (Table 1). A larger proportion of DOAC users reported a history of switching from another anticoagulant (42.9%) compared with only 9.5% of warfarin users (p < 0.0001). Patients who reported switching from warfarin to a DOAC were more likely to cite convenience (40.6% v. 19.3%, p < 0.0001) and drug-drug interactions (13% v. 5%, p = 0.016) as reasons for switching anticoagulants.

**Table 1:** Survey of 2217 patients taking anticoagulants for venous thromboembolism, comparing characteristics of DOAC and warfarin users

Characteristic	DOAC users (n = 969)	Warfarin users (n = 1248)	p-value
Age at time of survey			0.016
≤ 54 years	158 (16.3%)	226 (18.1%)	
55–64 years	210 (21.7%)	254 (20.4%)	
65–74 years	317 (32.7%)	352 (28.2%)	
75–84 years	220 (22.7%)	293 (23.5%)	
≥ 85 years	64 (6.6%)	123 (9.9%)	
Women	404 (41.7%)	551 (44.2%)	0.25
Race			0.17
White	738 (76.2%)	895 (71.7%)	
Black	88 (9.1%)	131 (10.5%)	
Asian or Pacific Islander	25 (2.6%)	32 (2.6%)	
Multiple or other	87 (9%)	136 (10.9%)	
Missing	31 (3.2%)	54 (4.3%)	
Ethnicity			0.41
Hispanic	94 (9.7%)	140 (11.2%)	
Non-Hispanic	849 (87.6%)	1069 (85.7%)	
Missing	26 (2.7%)	39 (3.1%)	
Preferred language			0.60
English	921 (95%)	1168 (93.6%)	
Spanish	17 (1.8%)	31 (2.5%)	
Other, specify	3 (0.3%)	7 (0.6%)	
Missing	23 (2.4%)	36 (2.9%)	
Multiple mark	5 (0.5%)	6 (0.5%)	
Highest level of education			< 0.0001
Less than HS Graduate	33 (3.4%)	42 (3.4%)	
12th grade, HS graduate or GED	101 (10.4%)	199 (15.9%)	
Some college or technical school	356 (36.7%)	511 (40.9%)	
Completed Bachelor degree	230 (23.7%)	250 (20%)	
Completed Graduate degree	212 (21.9%)	197 (15.8%)	
Unknown/missing	37 (3.8%)	49 (3.9%)	
Total household income			0.007
Under $15,000	39 (4%)	47 (3.8%)	
$ 15,000 to $25,000	52 (5.4%)	93 (7.5%)	
$25,001 to $35,000	56 (5.8%)	89 (7.1%)	
$35,001 to $50,000	69 (7.1%)	130 (10.4%)	
$50,001 to $65,000	82 (8.5%)	118 (9.5%)	
$65,001 to $80,000	94 (9.7%)	112 (9%)	
$80,001 to $100,000	93 (9.6%)	132 (10.6%)	
$100,001 to $150,000	150 (15.5%)	150 (12%)	
More than $150,000	123 (12.7%)	121 (9.7%)	
I do not want to answer this question	162 (16.7%)	185 (14.8%)	
Missing	49 (5.1%)	71 (5.7%)	
Marital status			0.50
Married	599 (61.8%)	731 (58.6%)	
Not married but in a committed relationship	38 (3.9%)	63 (5%)	
Widowed	97 (10%)	128 (10.3%)	
Single, divorced, or separated	201 (20.7%)	282 (22.6%)	
Missing	34 (3.5%)	44 (3.5%)	
Family history of DVT/PE	203 (20.9%)	239 (19.2%)	0.28
Year of index VTE			< 0.0001
2015	156 (16.1%)	462 (37%)	
2016	316 (32.6%)	483 (38.7%)	
2017	322 (33.2%)	231 (18.5%)	
2018	175 (18.1%)	72 (5.8%)	
Switched from one anticoagulant to another			< 0.0001
No	515 (53.1%)	1074 (86.1%)	
Yes	416 (42.9%)	119 (9.5%)	
Missing	10 (1%)	21 (1.7%)	
Do not know	28 (2.9%)	34 (2.7%)	
If switched anticoagulants, reasons for changing (can list more than one)			
Side effects	92 (22.1%)	29 (24.4%)	0.60
Convenience	169 (40.6%)	23 (19.3%)	< 0.0001
Diet or drug interactions	54 (13%)	6 (5%)	0.02
Cost	37 (8.9%)	10 (8.4%)	0.87
Difficulty with blood thinner control	76 (18.3%)	26 (21.8%)	0.38
I do not know	93 (22.4%)	36 (30.3%)	0.08
Sought medical assistance for bleeding problems while on anticoagulants			0.057
No	862 (89%)	1065 (85.3%)	
Yes	99 (10.2%)	162 (13%)	
Missing	4 (0.4%)	12 (1%)	
Do not know	4 (0.4%)	9 (0.7%)	

On unadjusted analysis, DOAC users were on average more satisfied than warfarin users with anticoagulant treatment, with both higher mean ACTS Burdens score (52.9 vs. 50.6, p < 0.0001) and higher ACTS Benefits score (10.4 vs. 10.1, p = 0.005, Table 2). The clinical effect size of these differences was small overall (0.29 for ACTS Burdens and 0.12 for ACTS Benefits).

**Table 2:** Anticoagulant treatment satisfaction of 2217 patients taking anticoagulants for venous thromboembolism, measured by the ACTS Burdens, ACTS Benefits, and ACTS Global Satisfaction scores

	DOAC users (n = 969)	Warfarin users (n = 1248)	Effect size^a^	p-value
ACTS Burden Scale (mean, SD)	52.9 (7.20)	50.6 (8.50)	0.29	< 0.0001
ACTS Burden Global (mean, SD)	4.2 (0.99)	4.0 (1.04)	0.15	0.0001
ACTS Benefit Scale (mean, SD)	10.4 (2.98)	10.1 (3.06)	0.12	0.0047
ACTS Benefit Global (mean, SD)	3.2 (1.24)	3.1 (1.24)	0.08	0.0412

Higher scores indicate greater treatment satisfaction

*SD* standard deviation

^a^An effect size of 0.20 is considered small, an effect size of 0.50 is moderate, and effect sizes of ≥ 0.80 are large

After multivariable adjustment, the difference in satisfaction between DOAC and warfarin users persisted, with DOAC users having higher mean ACTS Burdens (50.18 v. 48.01, p < 0.0001, Table 3) and ACTS Benefits scores (10.21 v. 9.84, p = 0.046, Table 4). The final models of treatment satisfaction were adjusted for age, gender, race, Hispanic ethnicity, household income, educational attainment, marital status, patient-reported history of bleeding, VTE type, year of diagnosis, whether the patient reported switching anticoagulants, and the hdPS. Several other factors were also associated with differences in treatment satisfaction. In particular, women, younger age, and a history of bleeding were associated with more perceived anticoagulant burden (Table 3), while a history of switching anticoagulants and prior bleeding were associated with lower perceived benefits (Table 4).

**Table 3:** Anticoagulant treatment burden among patients with venous thromboembolism: adjusted least mean ACTS Burdens score with 95% confidence intervals (CI), and P-values for the difference in means from multivariable general linear model*

Variable	Adjusted mean ACTS Burdens score	95% CI (low)	95% CI (high)	p-value
Treatment type				
Warfarin	48.01	46.80	49.23	Ref
DOAC	50.18	48.99	51.37	< 0.0001
Age at time of survey				
≤ 54	45.28	44.01	46.55	Ref
55–64	48.25	46.98	49.52	< 0.0001
65–74	49.73	48.48	50.97	< 0.0001
75–84	50.56	49.28	51.85	< 0.0001
≥ 85	51.67	50.07	53.27	< 0.0001
Gender				
Male	49.60	48.42	50.78	Ref
Female	48.60	47.44	49.75	0.0049
Race				
White	49.77	48.70	50.83	Ref
Asian or Pacific Islander	49.42	47.17	51.66	0.74
Black	49.34	47.87	50.81	0.47
Other/missing	47.87	46.58	49.15	0.0015
Ethnicity				
Not Hispanic or missing ethnicity	49.66	48.58	50.74	Ref
Hispanic	48.54	47.07	50.00	0.0901
Highest level of education				
Less than HS Graduate	48.16	46.04	50.28	Ref
12th grade, HS graduate or GED, some college or technical school	49.62	48.50	50.74	0.16
Completed Bachelor degree	49.79	48.54	51.04	0.13
Completed Graduate degree	48.83	47.53	50.12	0.55
Income				
≤ $25,000	48.39	46.97	49.81	Ref
$25,001 to $50,000	49.62	48.30	50.94	0.068
$50,001 to $100,000	49.73	48.50	50.96	0.0355
≥ $100,001	49.44	48.09	50.78	0.1345
Prefer not to answer	48.31	46.93	49.70	0.909
Marital status				
Single, divorced, widowed or separated	49.22	48.09	50.35	Ref
Married, or not married but in a committed relationship	48.98	47.75	50.21	0.56
VTE type				
Pulmonary embolism	48.31	47.28	49.34	
Lower extremity deep vein thrombosis	48.82	47.76	49.88	0.15
Upper extremity deep vein thrombosis	48.46	46.56	50.36	0.86
Other VTE (mesenteric venous thrombosis, other VTE, unknown VTE)	50.80	48.76	52.84	0.0096
Year of index VTE				
2015	49.58	48.34	50.82	Ref
2016	49.18	47.99	50.37	0.36
2017	48.50	47.25	49.75	0.03
2018	49.13	47.67	50.60	0.49
History of switching anticoagulants				
No	49.45	48.32	50.59	Ref
Yes	48.74	47.50	49.99	0.096
History of bleeding issue				
No	51.21	50.14	52.27	Ref
Yes	46.99	45.62	48.37	< 0.0001

Higher scores denote greater treatment satisfaction

*Model developed from 2211 survey respondents without missing data and included all listed covariates in addition to a high-dimensional propensity score (hdPS). The c-statistic for the hdPS was 0.858

**Table 4:** Anticoagulant treatment benefits among patients with venous thromboembolism: adjusted least mean ACTS Benefits score with 95% confidence intervals (CI), and P-values for the difference in means from multivariable general linear model*

Variable	Adjusted mean ACTS Benefits score	95% CI (low)	95% CI (high)	p-value
Treatment type				
Warfarin	9.84	9.34	10.33	Ref
DOAC	10.21	9.73	10.69	0.046
Age at time of survey				
≤ 54	9.91	9.40	10.43	Ref
55–64	10.03	9.52	10.54	0.60
65–74	10.26	9.75	10.76	0.10
75–84	9.86	9.34	10.38	0.81
≥ 85	10.06	9.41	10.71	0.63
Gender				
Male	10.12	9.64	10.60	Ref
Female	10.07	9.61	10.52	0.16
Race				
White	10.05	9.61	10.48	Ref
Asian or Pacific Islander	10.38	9.47	11.29	0.44
Black	9.67	9.08	10.27	0.12
Other or missing race	10.00	9.47	10.52	0.83
Ethnicity				
Not Hispanic or missing ethnicity	9.89	9.45	10.33	Ref
Hispanic	10.16	9.56	10.76	0.31
Highest level of education				
Less than HS Graduate	9.48	8.62	10.34	Ref
12th grade, HS graduate or GED, some college or technical school	10.07	9.61	10.52	0.16
Completed Bachelor degree	10.33	9.83	10.84	0.054
Completed Graduate degree	10.21	9.69	10.73	0.10
Income				
≤ $25,000	9.71	9.13	10.28	Ref
$25,001 to $50,000	10.09	9.56	10.63	0.16
$50,001 to $100,000	10.20	9.70	10.70	0.058
≥ $100,001	10.22	9.67	10.76	0.070
Prefer not to answer	9.90	9.34	10.46	0.51
Marital status				
Single, divorced, widowed or separated	9.89	9.43	10.35	
Married, or not married but in a committed relationship	10.15	9.65	10.65	0.11
VTE type				
Pulmonary embolism	9.96	9.55	10.38	Ref
Lower extremity deep vein thrombosis	9.77	9.34	10.20	0.17
Other VTE (mesenteric venous thrombosis, other VTE, unknown VTE)	10.03	9.25	10.80	0.86
Upper extremity deep vein thrombosis	10.33	9.51	11.16	0.34
Year of index VTE				
2015	10.32	9.82	10.83	Ref
2016	9.91	9.43	10.40	0.019
2017	10.04	9.53	10.55	0.16
2018	9.81	9.22	10.41	0.051
History of switching anticoagulant				
No	10.20	9.74	10.66	Ref
Yes	9.85	9.34	10.35	0.043
History of bleeding issue				
No	10.39	9.96	10.82	Ref
Yes	9.66	9.10	10.21	0.0006

*Developed on 2207 survey respondents without missing data and included all listed covariates in addition to a high-dimensional propensity score (hdPS). The c-statistic for the hdPS was 0.861

Higher scores denote greater treatment satisfaction

In the subgroup analysis of patients who did not report a history of switching anticoagulants, patients taking DOACs continued to have a higher mean ACTS Burdens Score (50.84 v. 48.37, p = 0.0016), but the difference in mean ACTS Benefits Score did not remain statistically different (Online Appendix Tables A and B).

## Discussion

In this study of 2217 people taking anticoagulants for VTE, patients taking DOACs reported greater anticoagulant treatment satisfaction than did warfarin users, although the magnitude of the difference was small. Few studies have directly compared treatment satisfaction between DOACs and warfarin for VTE in non-trial settings. The initial development and validation of the ACTS Score were based on the EINSTEIN DVT and EINSTEIN PE randomized trials and measured treatment satisfaction only up to 12 months after drug initiation [12, 13]. The EINSTEIN studies found that rivaroxaban was associated with greater treatment satisfaction than enoxaparin/vitamin K antagonists for the initial treatment of thromboembolism. Subsequently, a sub-analysis of the observational XArelto for Long-term and Initial Anticoagulation in venous thromboembolism (XALIA) study compared treatment satisfaction between rivaroxaban or enoxaparin/vitamin K antagonists for DVT [5]. After a median ~ 180 days of treatment, rivaroxaban was associated with less burden. In these studies of rivaroxaban, the mean ACTS Burdens score ranged from 55.2 to 56.1 and the mean ACTS Benefits score ranged from 11.7 to 12.1. Interestingly, these scores are higher than the mean scores found in our study population. Our study was conducted in a diverse population, based in the United States (as opposed to a primarily European population) and included patients who were beyond the initial treatment phase of anticoagulation. Patients enrolled in clinical trial settings may also be more highly selected and closely followed than patients in unselected real-world settings, highlighting the importance of conducting evaluations of actual clinical practice.

The primary objective of this study was to compare treatment satisfaction between DOAC and warfarin users. We did, in addition, find differences in treatment satisfaction among several patient sub-groups. Specifically, women and people of younger ages perceived more burden associated with anticoagulant treatment, regardless of type of therapy. A history of needing to seek medical attention for anticoagulant-related bleeding was associated with more perceived burden and lower benefits.

Our study had several limitations. As an observational study of clinical care, patients were not randomly assigned to take warfarin or DOACs. Although we applied high dimensional propensity score approaches to better balance the two groups, it is possible that treatment satisfaction may have been influenced by factors leading to preferential use of one anticoagulant over another, such framing of medications by individual clinicians, intercurrent health events, and impact on lifestyle. Although our survey was large, with over 2000 responses, some people who were eligible to participate either declined or did not respond and it is possible that the experiences of people who answered the survey do not fully represent the perspectives of the overall population.

In summary, this large survey of community-based patients taking anticoagulants for VTE found greater treatment satisfaction among patients taking DOACs, although the differences were small clinically. Our study was not designed to determine whether there is an association between treatment satisfaction and adherence to anticoagulant treatment. However, other studies have found that increased satisfaction has positive impact on medication compliance and persistence [14]. Findings from this study can help clinicians inform patients of the comparative differences between these two anticoagulant options. If all other factors are equal, such as medical appropriateness and cost, then patients may prefer being on a DOAC.

## Supplementary Information

Below is the link to the electronic supplementary material.

Supplementary file1 (DOCX 38 kb)

## Data Availability

Upon request.

## Code availability

Upon request.
