# Supplementary material for: Anticoagulant treatment satisfaction with warfarin and direct oral anticoagulants for venous thromboembolism
Source: J Thromb Thrombolysis. 2021 Apr 8;52(4):1101–9. doi: 10.1007/s11239-021-02437-z (PMC8605968; doi:10.1007/s11239-021-02437-z)
Supplement: Supplementary file 1 — Supplementary file1 (DOCX 38 kb) [file 11239_2021_2437_MOESM1_ESM.docx]

**Supplemental Material**

**Appendix Table A. Anticoagulant Treatment Burden Among Subjects Without a Reported History of Switching Anticoagulants:** Adjusted Least Mean ACTS Burdens Score with 95% Confidence Intervals (CI), and P-values for Difference in Means from Multivariable General Linear Model*. Higher scores denote greater treatment satisfaction.

| Variable | Adjusted Mean ACTS Burdens Score | 95% CI (low) | 95% CI (high) | p-value |
| --- | --- | --- | --- | --- |
| Treatment type |  |  |  |  |
| Warfarin | 48.37 | 46.99 | 49.75 | ref |
| DOAC | 50.84 | 49.18 | 52.51 | 0.0016 |
| Race |  |  |  |  |
| White/European | 50.45 | 49.17 | 51.73 | ref |
| Asian/Pacific Islander | 49.80 | 47.19 | 52.41 | 0.60 |
| Black/African American | 50.36 | 48.61 | 52.10 | 0.89 |
| Other/Unknown | 47.82 | 46.28 | 49.36 | 0.0002 |
| Ethnicity |  |  |  |  |
| Not Hispanic, Unknown | 49.84 | 48.54 | 51.14 | ref |
| Hispanic | 49.37 | 47.62 | 51.12 | 0.56 |
| Age category at survey |  |  |  |  |
| ≤ 54 | 45.78 | 44.29 | 47.27 | ref |
| 55-64 | 48.54 | 47.03 | 50.05 | <.0001 |
| 65-74 | 50.09 | 48.61 | 51.57 | <.0001 |
| 75-84 | 51.10 | 49.58 | 52.63 | <.0001 |
| ≥85 | 52.52 | 50.64 | 54.39 | <.0001 |
| Gender |  |  |  |  |
| Male | 50.10 | 48.71 | 51.49 | ref |
| Female | 49.11 | 47.73 | 50.49 | 0.017 |
| Highest level of education |  |  |  |  |
| Less than HS Graduate | 48.14 | 45.62 | 50.66 | ref |
| 12th grade, HS graduate or GED,  Some college or technical school | 50.17 | 48.85 | 51.49 | 0.10 |
| Completed Bachelor degree | 50.42 | 48.93 | 51.92 | 0.08 |
| Completed Graduate degree | 49.69 | 48.13 | 51.26 | 0.24 |
| Income |  |  |  |  |
| ≤ $25,000 | 48.79 | 47.09 | 50.49 | ref |
| $25,001 to $50,000 | 50.25 | 48.69 | 51.81 | 0.064 |
| $50,001 to $100,000 | 50.10 | 48.63 | 51.56 | 0.08 |
| ≥$100,001 | 50.28 | 48.70 | 51.87 | 0.073 |
| Prefer not to answer | 48.61 | 46.98 | 50.23 | 0.83 |
| Marital Status |  |  |  |  |
| Single, Divorced, Widowed, or  separated | 49.74 | 48.39 | 51.10 | ref |
| Married or Not married but in a  committed relationship | 49.47 | 48.01 | 50.92 | 0.56 |
| History of bleeding issue |  |  |  |  |
| No | 51.62 | 50.38 | 52.87 | ref |
| Yes | 47.59 | 45.92 | 49.26 | <.0001 |
| VTE Type |  |  |  |  |
| Pulmonary Embolism | 48.83 | 47.61 | 50.06 | ref |
| Lower Extremity Deep Vein  Thrombosis | 49.17 | 47.90 | 50.44 | 0.42 |
| Other VTE (Mesenteric Venous  Thrombosis, Other VTE, Unknown  VTE) | 48.80 | 46.61 | 50.99 | 0.97 |
| Upper Extremity Deep Vein  Thrombosis | 51.62 | 49.20 | 54.04 | 0.014 |
| Year of Index VTE |  |  |  |  |
| 2015 | 50.10 | 48.61 | 51.59 | ref |
| 2016 | 49.76 | 48.33 | 51.20 | 0.50 |
| 2017 | 49.10 | 47.60 | 50.59 | 0.092 |
| 2018 | 49.46 | 47.80 | 51.13 | 0.39 |

*Developed on 1587 survey respondents without missing data and included all listed covariates in addition to a high-dimensional propensity score (hdPS). The c-statistic for the hdPS was 0.969.

**Appendix Table B. Anticoagulant Treatment Benefits Among Subjects Without a Reported History of Switching Anticoagulants:** Adjusted Least Mean ACTS Benefits Score with 95% Confidence Intervals (CI), and P-values for Difference in Means from Multivariable General Linear Model*. Higher scores denote greater treatment satisfaction.

| Variable | Adjusted Mean ACTS Benefits Score | 95% CI (low) | 95% CI (high) | p-value |
| --- | --- | --- | --- | --- |
| Treatment type |  |  |  |  |
| Warfarin | 9.89 | 9.32 | 10.46 | ref |
| DOAC | 10.01 | 9.31 | 10.71 | 0.74 |
| Race |  |  |  |  |
| White/European | 10.07 | 9.56 | 10.59 | ref |
| Asian/Pacific Islander | 10.20 | 9.15 | 11.25 | 0.80 |
| Black/African American | 9.50 | 8.80 | 10.21 | 0.040 |
| Other/Unknown | 10.04 | 9.41 | 10.66 | 0.90 |
| Ethnicity |  |  |  |  |
| Not Hispanic, Unknown | 9.85 | 9.33 | 10.37 | ref |
| Hispanic | 10.05 | 9.35 | 10.76 | 0.53 |
| Age category at survey |  |  |  |  |
| ≤ 54 | 9.96 | 9.36 | 10.56 | ref |
| 55-64 | 9.93 | 9.32 | 10.54 | 0.91 |
| 65-74 | 10.27 | 9.67 | 10.87 | 0.20 |
| 75-84 | 9.68 | 9.07 | 10.30 | 0.29 |
| ≥85 | 9.92 | 9.16 | 10.68 | 0.91 |
| Gender |  |  |  |  |
| Male | 10.07 | 9.51 | 10.63 | ref |
| Female | 9.84 | 9.28 | 10.40 | 0.18 |
| Highest level of education |  |  |  |  |
| Less than HS Graduate | 9.28 | 8.27 | 10.28 | ref |
| 12th grade, HS graduate or GED,  Some college or technical school | 9.97 | 9.43 | 10.50 | 0.17 |
| Completed Bachelor degree | 10.38 | 9.78 | 10.99 | 0.03 |
| Completed Graduate degree | 10.18 | 9.55 | 10.82 | 0.089 |
| Income |  |  |  |  |
| ≤ $25,000 | 9.78 | 9.09 | 10.47 | ref |
| $25,001 to $50,000 | 9.99 | 9.37 | 10.62 | 0.50 |
| $50,001 to $100,000 | 10.14 | 9.55 | 10.73 | 0.23 |
| ≥$100,001 | 10.18 | 9.54 | 10.82 | 0.24 |
| Prefer not to answer | 9.67 | 9.02 | 10.33 | 0.76 |
| Marital Status |  |  |  |  |
| Single, Divorced, Widowed, or  separated | 9.82 | 9.27 | 10.36 | ref |
| Married or Not married but in a  committed relationship | 10.09 | 9.50 | 10.68 | 0.16 |
| History of bleeding issue |  |  |  |  |
| No | 10.35 | 9.85 | 10.86 | ref |
| Yes | 9.55 | 8.88 | 10.22 | 0.0021 |
| VTE Type |  |  |  |  |
| Pulmonary Embolism | 9.97 | 9.48 | 10.47 |  |
| Lower Extremity Deep Vein  Thrombosis | 9.69 | 9.18 | 10.21 | 0.10 |
| Other VTE (Mesenteric Venous  Thrombosis, Other VTE, Unknown  VTE) | 9.78 | 8.89 | 10.67 | 0.64 |
| Upper Extremity Deep Vein  Thrombosis | 10.37 | 9.39 | 11.34 | 0.39 |
| Year of Index VTE |  |  |  |  |
| 2015 | 10.20 | 9.60 | 10.80 | ref |
| 2016 | 9.87 | 9.29 | 10.45 | 0.11 |
| 2017 | 9.95 | 9.35 | 10.55 | 0.30 |
| 2018 | 9.79 | 9.11 | 10.46 | 0.16 |

*Developed on 1583 survey respondents without missing data and included all listed covariates in addition to a high-dimensional propensity score (hdPS). The c-statistic for the hdPS was 0.980.
